# Reactive Hypoglycemia: A Trigger for Nutrient-Induced Endocrine and Metabolic Responses in Polycystic Ovary Syndrome

**DOI:** 10.3390/jcm12237252

**Published:** 2023-11-23

**Authors:** Sidika E. Karakas

**Affiliations:** 1 Division of Endocrinology, Diabetes and Metabolism, School of Medicine, University of California Davis, Davis, CA 95616, USA; sekarakas@ucdavis.edu; 2 University of California Medical Center Sacramento, Sacramento, CA 95817, USA

**Keywords:** reactive hypoglycemia, postprandial hypoglycemia, polycystic ovary syndrome, protein supplements, whey protein, weight loss, adrenal steroids, ghrelin

## Abstract

Polycystic ovary syndrome (PCOS) is an insulin-resistant state compensated for by the body via hyperinsulinemia. More than 50% of women with PCOS are obese and/or have metabolic syndrome. Weight loss improves both metabolic and reproductive outcomes. Energy/caloric content as well as the nutrient composition of one’s diet may also be important. This article will present a series of studies from our research comparing the effects of dietary protein vs. simple carbohydrates (CHOs). The results of the acute challenge studies demonstrate that simple CHO intake causes reactive hypoglycemia in one third of women with PCOS, especially among obese and insulin-resistant individuals. Symptoms of hypoglycemia are associated with secretion of cortisol and adrenal androgens. Simple CHOs suppress the hunger signal ghrelin for a shorter period. During weight loss, women who receive protein supplementation achieve more significant weight and fat mass losses. The amino acid compositions of the protein supplements do not affect the improvements in weight and insulin resistance. It is plausible that simple CHO intake leads to weight gain, or interferes with weight loss, by causing reactive hypoglycemia, triggering adrenal steroid secretion and thus leading to snacking. Since obese women with PCOS are more susceptible to reactive hypoglycemia, a vicious cycle is established. Restriction of simple CHOs may break this cycle.

## 1. Introduction

Characteristic features of polycystic ovary syndrome (PCOS) include oligomenorrhea/amenorrhea/anovulation, hyperandrogenemia and cystic ovaries. In addition, women with PCOS have insulin resistance and hyperinsulinemia even in the absence of obesity. In the USA, 69% of women with PCOS are obese and 64% have metabolic syndrome [1,2]. Weight loss improves both metabolic and reproductive outcomes in PCOS [3,4,5,6]. Our earlier research in women without PCOS demonstrated that replacement of dietary fat with carbohydrates (CHOs) can lead to weight loss in free-living conditions [7]. However, those women who do not lose weight on a low-fat/high-CHO diet experience worsening of dyslipidemia and a rise in inflammatory risk factors [8]. This article will summarize the results of our nutrition research in women with PCOS. Potential links between the nutrient composition of their diets and the changes in anthropometric and metabolic outcomes will be discussed. A unifying conceptual framework for a dietary approach to PCOS will be presented.

A notable finding of our studies was that simple CHO intake was associated with reactive hypoglycemia in a significant number of PCOS patients [9,10] and triggered adrenal steroid secretion. Hypoglycemia is defined as low blood glucose concentrations that can cause harm to an individual. Glucose levels below 70 mg/dL are considered mild, and those below 54 mg/dL are considered serious hypoglycemia. Mild hypoglycemia leads to adrenergic symptoms such as tremors, palpitations, sweating, hunger and paresthesia. Serious hypoglycemia causes additional neuroglycopenic symptoms such as headache, dizziness, confusion, amnesia, seizure and coma [11].

Hypoglycemia occurring within four hours after a meal is called reactive or postprandial hypoglycemia [12]. This condition is frequently undermined by the medical community for several reasons: Hypoglycemic symptoms can occur without biochemical evidence of low blood glucose. There is no accurate method of diagnosing postprandial hypoglycemia. The oral glucose tolerance test (OGTT) overestimates the problem since 10% of individuals tested with an extended (5 h) OGTT develop blood glucose below 50 mg/dL. A mixed-meal challenge can elicit neuroglycopenic symptoms in the absence of hypoglycemia. Because of these limitations, reactive hypoglycemia is considered significant when it develops after bariatric surgery, significant alcohol intake or in the rare case of hereditary fructose intolerance or insulinoma.

The goal of our research was to determine the optimal weight loss diet for women with PCOS. All the studies presented here were approved by the Institutional Review Boards, peer-reviewed and published. We addressed the following questions:

1. Does replacement of dietary fat with CHOs vs. protein influence the amount of weight loss and body composition?
2. What are the acute metabolic and endocrine effects of CHO and protein intake on PCOS?
3. Do the amino acid compositions of dietary proteins affect weight loss and/or insulin resistance?

### 1.1. Comparing Low-Fat/High-CHO vs. Low-Fat/High-Protein Diets during Weight Loss in Women with PCOS

#### Acute Effects of Simple CHOs vs. Protein

Thirty-three women who fulfilled the National Institutes of Health criteria for PCOS participated in this 2-month-long, free-living, randomized, single-blinded study [13]. To achieve a final energy reduction of 450 kcal/day, their daily energy intake was reduced by 700 kcal, and a 240 kcal supplement containing either whey protein (WP) or simple CHOs was added. The powdered supplements contained either sugar-free WP isolate (96% pure) or simple sugars (glucose plus maltose) and were packaged in individual, identical-looking pouches to allow blinding. Because whey is naturally calcium-enriched, and calcium can independently promote weight loss, the calcium contents of the supplements were equalized by adding tricalcium phosphate to the carbohydrate supplement. Whey protein supplement was sweetened by adding a non-caloric sugar substitute; both supplements were similarly flavored. Most participants consumed the supplements as a partial meal replacement for breakfast.

**Figure 1:**
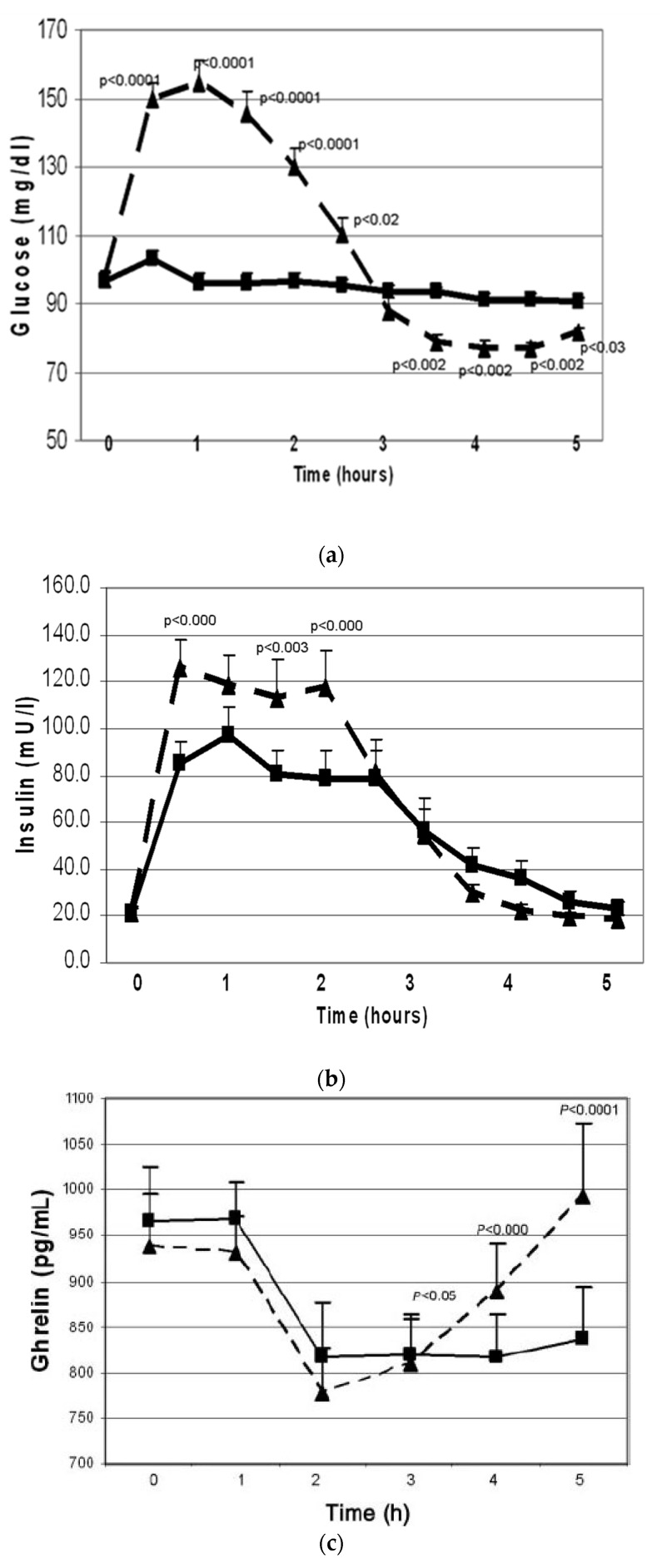
Changes in plasma glucose *(***a**), insulin (**b**) and total plasma ghrelin (**c**) (mean ± SEM) during oral glucose tolerance (*n* = 28, dashed line) and protein challenge (*n* = 23, solid line) tests. Differences between responses to the two treatments are expressed through the treatment-by-time interaction effect. Overall, the interaction effect was highly significant (*p* < 0.001). Key timepoint *p* values are shown on the graph. All tests were based on the Wald test performed by using a linear mixed model applied to all available data (from reference [10]).

First, the effects of oral whey protein (WP) vs. simple sugar intakes were examined during 5 h challenge studies [10]. Even though WP did not change plasma glucose concentrations (Figure 1a), it increased plasma insulin 5-fold (Figure 1b), indicating that WP is a potent insulin secretagogue. Another significant difference was seen in plasma ghrelin levels. Ghrelin is a gastrointestinal hormone secreted in the stomach and duodenum. It is a “hunger signal”. It is suppressed by food intake and rises to signal hunger when the stomach empties. While both simple sugar and WP similarly suppressed ghrelin, after simple sugar intake, ghrelin started to rise after two hours, whereas after WP intake, ghrelin remained suppressed throughout the five-hour test (Figure 1c). This observation suggested that protein intake may provide satiety for a longer period as compared to simple sugar.

### 1.2. Postprandial Hypoglycemia after Simple Sugar vs. Protein Intake

**Figure 2:**
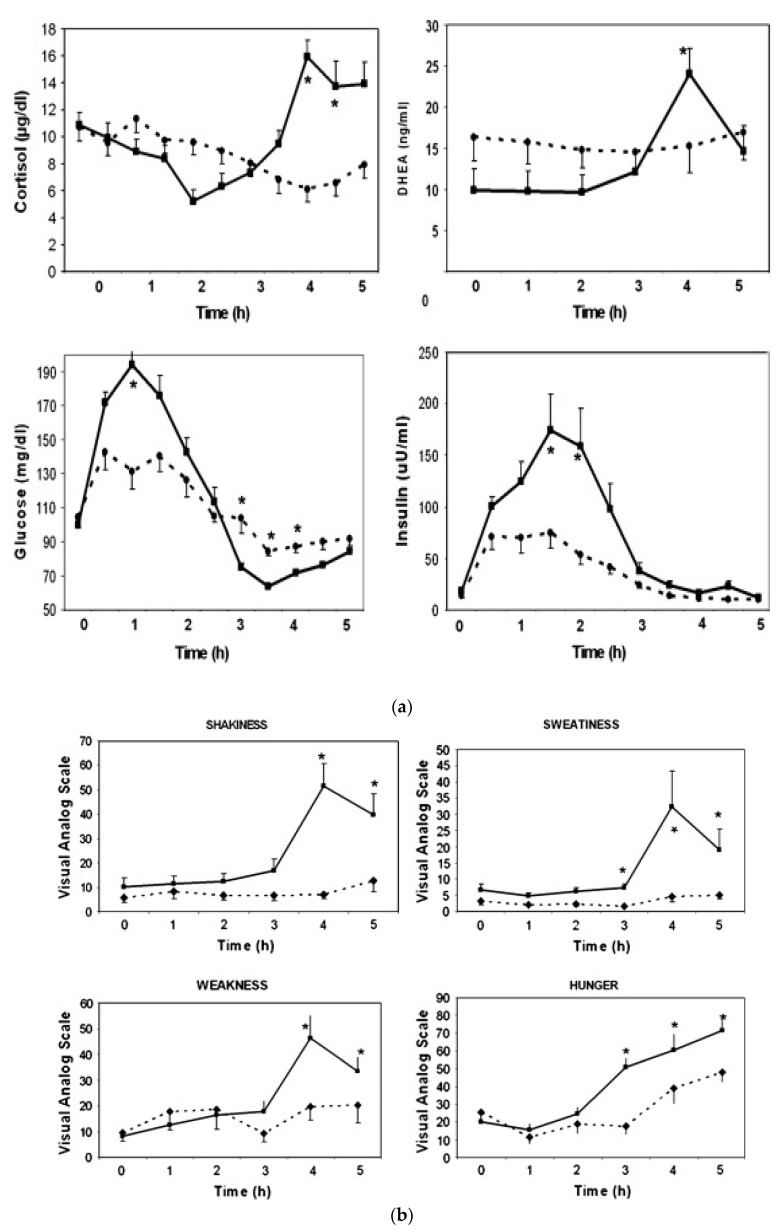
Changes in cortisol and DHEA, glucose and insulin (**a**) and clinical symptoms (**b**) in responders (*n* = 9, solid line) vs. non-responders (*n* = 10, dashed line) during oral glucose tolerance test (mean ± SEM, *; *p* < 0.05 when responders are compared to non-responders) (from reference [9]).

During the previous studies, a significant number of PCOS patients developed the symptoms of hypoglycemia after simple sugar consumption. It is well known that hypoglycemia stimulates the secretion of several pituitary hormones, including ACTH, and triggers the secretion of adrenal hormones [14,15]. Therefore, the next study focused on hypoglycemic symptoms as they relate to adrenal function [9]. Since the standard test used to evaluate this is the 5 h oral glucose test (OGTT), responses to glucose vs. WP were compared. The adrenal response was defined based on cortisol changes during the OGTT. Subjects who had a minimum increase of 7.2 μg/dL (200 nM) in cortisol were defined as “responders” because such a response is considered positive during cortrysin stimulation testing. In total, 9 subjects had a 10.7 ± 1.0 µg/dL increase in cortisol (responders); 10 subjects had a 3.5 ± 0.6 µg/dL decrease (non-responders); and 11 subjects had an intermediate response of a 4.3 ± 1.0 µg/dL increase (*p* < 0.0001). The changes in DHEA concentrations followed a similar pattern: Δ = 14.4 ± 1.7 ng/mL in the responders, Δ = 0.4 ± 0.9 ng/mL in the non-responders and Δ = 3.6 ± 1.2 ng/mL in the intermediates (*p* = 0.0003) (Figure 2a).

Responders had higher glucose levels at 1 h (194 ± 13 vs. 131 ± 12 mg/dL, *p* < 0.05) but lower nadir glucose levels later during the test (61.4 ± 2.2 vs. 70.2 ± 2.3 mg/dL, *p* = 0.0002). Responders also had a higher insulin response at 2 h as compared to non-responders (159 ± 31 vs. 54 ± 29 mU/mL, *p* < 0.05) (Figure 2a).

The key clinical symptoms related to the autonomic response, neuroglycopenia and malaise (sweating, shaking, hunger, weakness, confusion, drowsiness, behavior, speech difficulty, incoordination, nausea and headache) were monitored using the Hypoglycemia Symptoms Logs program developed in collaboration with William Horn and Nancy Keim, PhD [9]. The symptoms were recorded hourly on a 0–100 scale on hand-held tablets. The data were transferred from the tablets to an Excel spreadsheet for analysis and were plotted against time. When responders were compared to non-responders, the symptoms diverged at the 3rd hour. The responders had higher scores in shakiness, sweatiness, weakness and hunger (Figure 2b).

When the baseline characteristics of responders were compared to those of non-responders, the responders were more obese (BMI: 37.0 ± 1.6 vs. 31.7 ± 1.8 kg/m^2^, *p* < 0.05) and had higher serum leptin levels (28.9 ± 1.7 vs. 24.1 ± 1.1 ng/mL, *p* < 0.03). The responders also had lower and sex hormone-binding globulin (SHBG) levels (33.9 ± 3.1 vs. 58.6 ± 6.7 nmol/L, *p* = 0.022).

These results indicate that one third of the women with PCOS developed physiologically significant reactive hypoglycemia stimulating adrenal steroid secretion. These patients were more obese and insulin-resistant as compared to those who did not develop hypoglycemia.

### 1.3. Effects of Dietary CHOs vs. Protein on Anthropometric Outcomes during Weight Loss Intervention

These studies compared the effects of WP vs. simple CHOs on changes in weight and body composition [13]. Twenty-four women who fulfilled the National Institutes of Health criteria for PCOS completed the 2-month, free-living, randomized, single-blinded study. Habitual energy intake was reduced by 700 kcal, and a 240 kcal supplement containing either WP or simple CHOs was added. The final energy restriction was −450 kcal/day. After randomization, 13 participants first received the simple CHO supplement, and 11 participants received the WP supplement. Seven-day food records were analyzed using NutritionistPro (v7.9, Redmond, WA, USA).

The baseline energy intakes were similar (1947 ± 166 kcal/d in the WP; 1770 ± 157 kcal/d in the simple CHO groups). Energy intake decreased similarly (by 476 and 400 kcal/day, respectively). Protein intake increased from 20% to 33% in the WP group and decreased from 22% to 17% in the simple CHO group. Body composition was determined using electrical bioimpedance.

**Figure 3:**
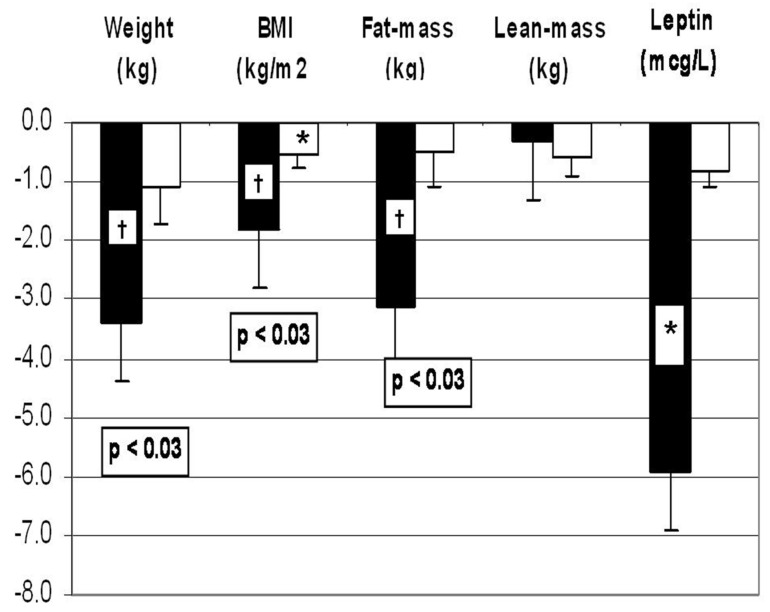
Effects of whey protein (WP: black columns) vs. simple carbohydrate (CHO: white columns) supplements on weight, body mass index (BMI), fat mass and plasma leptin concentrations during two months of weight loss intervention. *: *p* < 0.05 and †: *p* < 0.01 when compared to before-weight-loss baseline; numeric *p* values indicate the differences between the WP vs. simple CHO supplements. Changes in the variables over time were analyzed using mixed-model analysis of variance methods. Post hoc comparisons between timepoints were conducted using paired *t*-tests. A significance level of 0.05 was used to determine statistical significance of observed differences. Post hoc comparisons between the treatment groups with respect to contemporaneous changes in the same response measures were based on two-sample *t*-tests. From reference [13]).

Those receiving WP lost more weight (−3.3 +/−0.8 kg vs. −1.1 +/−0.6 kg, *p* < 0.03) and more fat mass (−3.1 +/−0.9 kg vs. −0.5 +/−0.6 kg, *p* < 0.03). Serum leptin was determined to be an independent measure of fat mass, and it decreased from 37.2 ± 3.9 µg/L to 31.3 ± 3.4 µg/L in the WP group but did not change in the simple CHO group (Figure 3). The effects of weight loss on the insulin resistance parameters, endocrine hormones and plasma lipids are shown on Table 1.

Whey protein recipients had significantly larger decreases in serum cholesterol (−33.0 +/−8.4 mg/dL vs. −2.3 +/−6.8 mg/dL), high-density lipoprotein cholesterol (−4.5 +/−1.3 mg/dL vs. −0.4 +/−1.3 mg/dL) and apoprotein B (−20 +/−5 mg/dL vs. 3 +/−5 mg/dL) as compared to the simple CHO group (Table 1).

These results indicate that women with PCOS may lose more weight and fat mass on a hypocaloric high-protein diet as compared to those on a high-simple-CHO diet.

### 1.4. Effects of Amino Acid Composition of Dietary Protein on Weight Loss and Metabolic Parameters in Women with PCOS

Whey protein comprises 60% essential amino acids (EAAs) and 23% branched-chain amino acids (BCAAs) [16]. The literature-reported measurements of plasma metabolome indicated that plasma BCAA concentrations correlate with insulin resistance [17,18]. Since PCOS and metabolic syndrome are insulin-resistant states, it is important to determine whether WP, a rich source of BCAAs, can exacerbate insulin resistance. To evaluate this possibility, WP was compared to gelatin (a form of collagen) during weight loss in women with metabolic syndrome. Gelatin differs from WP significantly in its AA content [19]. It is an incomplete protein missing the essential AA tryptophan, while it is enriched with proline and hydroxyproline. Whey protein contains three times more BCAAs as compared to gelatin.

In an 8-week double-blinded, placebo-controlled, randomized weight loss intervention, 29 women with metabolic syndrome received either gelatin-based or WP-based supplements (Glanbia, Inc., Twin Falls, ID, USA), 20 g/day. The metabolome of 27 participants (WP: *n* = 16) and (gelatin: *n* = 11) was investigated at the beginning and at the end of the intervention using GC–time-of-flight mass spectrometry [20].

Before the intervention, plasma BCAA levels correlated with the homeostasis model assessment of insulin resistance (HOMA) (r = 0.52, 0.43 and 0.49 for Leu, Ile and Val, respectively; all *p* < 0.05). However, after the weight loss intervention, these correlations disappeared. There was no difference in plasma abundances (reported as quantifier ion peak height ÷ 100) of BCAAs between the WP and gelatin supplementation groups (Ile: gelatin: 637 ± 18 vs. WP: 744 ± 65), (Leu: gelatin: 1210 ± 33; WP: 1380 ± 79) and (Val: gelatin: 2080 ± 59; WP: group: 2510 ± 230).

These findings suggest the composition of dietary protein did not affect the anthropometric or metabolic outcomes. Whey protein supplementation did not cause insulin resistance when compared to gelatin. Weight loss was the most important determinant of insulin resistance.

## 2. Discussion and a Unifying Hypothesis

Our studies indicated that (Table 2):

1. One third of the women with PCOS developed physiologically significant reactive hypoglycemia after simple sugar intake and secretion of cortisol and adrenal androgens.
2. Adrenal steroid secretion coincided with hypoglycemic symptoms.
3. Whey protein intake stimulated insulin secretion but did not cause hypoglycemia.
4. Whey protein supplementation suppressed the hunger signal ghrelin for a longer period as compared to simple CHO supplement.
5. A weight loss diet containing a WP supplement was associated with greater weight loss and fat mass loss and a decrease in leptin when compared to the diet containing a simple CHO supplement.
6. When the WP supplement was compared to the gelatin supplement, there was no difference in the amount of weight loss or the improvement in insulin sensitivity, despite the lower essential AA- and BCAA content of gelatin.

The literature supports that women with PCOS are susceptible to reactive hypoglycemia. Altuntas et al. tested 64 lean subjects with PCOS with an extended OGTT and reported the prevalence of reactive hypoglycemia to be 50% [21]. Mumm et al. compared 88 women with PCOS to 34 age- and BMI-matched controls using a 5 h OGTT [22]. Seventeen percent of the women with PCOS but none of the controls developed hypoglycemia. Obese women with PCOS who developed reactive hypoglycemia had a higher cumulative insulin response as compared to those who did not develop hypoglycemia.

It is well known that hypoglycemia stimulates the secretion of several pituitary hormones including ACTH [15]. Insulin-induced hypoglycemia is used to test central stimulation of adrenal function [14]. It is not known, however, whether reactive hypoglycemia can trigger adrenal hormone secretion as well. The literature suggests that women with PCOS may respond to hypoglycemia differently than control women. Sam et al. compared 10 women with PCOS to 9 age-, BMI- and ethnicity-matched controls using hypoglycemic clamp and found that women with PCOS had a three-fold higher glucagon response [23]. The other counter-regulatory hormones, such as growth hormone and cortisol, did not show differential responses. Gennarelli et al. used insulin-induced hypoglycemia to compare the counter-regulatory hormones in women with PCOS vs. control women [24]. Obese women with PCOS were less symptomatic and had a blunted noradrenaline response as compared to the obese controls. Lean women with PCOS had a greater increase in growth hormone as compared to lean controls.

Our studies focused on the clinical presentation of hypoglycemia. We showed that one third of women with PCOS developed hypoglycemic symptoms during the 5 h OGTT. The symptomatic patients had lower nadir glucose levels and secreted cortisol and adrenal androgens while experiencing symptoms. They were also more obese and insulin-resistant than the asymptomatic women with PCOS. The link between hypoglycemic symptoms and adrenal hormone secretion indicates that the clinical symptoms are important clues pointing to the triggering of the adrenals. The link between hypoglycemic symptoms and obesity suggests that hypoglycemia may alter eating/snacking behavior, as observed by Kishimoto in men with subclinical hypoglycemia [25].

Since PCOS is an insulin-resistant state and a significant number of patients may have obesity and metabolic syndrome, several studies attempted to identify the optimal diet for weight loss and maintenance in PCOS. We found that a high-protein diet containing WP was superior to a high-CHO diet in achieving weight loss and fat mass loss. Studies of plasma metabolome by Ooi et al. showed that branched-chain AA supplementation may increase fat oxidation [26], offering a potential mechanism for our observation. A meta-analysis including 24 studies by Wycherley et al. compared energy-restricted high-protein/low-fat vs. standard-protein weight loss diets and found that high-protein diets caused more significant weight loss and fat mass loss and lowered plasma triglyceride levels more than the standard-protein diets [27]. There were no differences in changes in plasma insulin and other lipids. Even though all these studies included obese, insulin-resistant patients, only two focused on PCOS. One of these was our study, which, as summarized earlier, found favorable effects of a high-protein diet [13]. The other study, which was conducted by Stames et al., did not find any difference between the high-protein vs. high-CHO diets [28]. Moran et al. reviewed the results of five studies in 137 women with PCOS [29]. The differences in study populations and dietary interventions did not permit a meta-analysis. It appeared that a monounsaturated-fat-enriched diet caused greater weight loss. Low-glycemic-index and/or low-CHO diets improved menstrual regularity and quality of life and elicited greater reductions in insulin resistance, fibrinogen and total as well as high-density lipoprotein cholesterol [30,31]. A high-protein diet improved depression and self-esteem [32], whereas a high-CHO diet increased the free androgen index. Most importantly, regardless of dietary composition, weight loss improved the presentation of PCOS. Sorensen et al. compared high-protein vs. standard-protein diets in a 6-month study of 27 women with PCOS and reported greater weight and fat mass losses with the high-protein diet [33].

Our initial high-protein weight loss studies used WP supplementation. Whey protein contains high amounts of essential AAs and BCAAs. Studies of the plasma metabolome indicated that plasma BCAAs correlated with insulin resistance [17,18]. Therefore, we compared WP to gelatin, a protein which is relatively poor in BCAAs; there was no difference in weight or fat mass loss or the change in insulin sensitivity [20]. It appeared that the protein content of the diet but not the AA composition of the protein was an important factor for weight loss.

In clinical practice, symptoms suggesting reactive hypoglycemia occur mostly during mid-morning and/or mid-afternoon and may include “getting hungry; craving sugar/carbohydrate”, “losing concentration/getting sleepy” and “developing headaches/sweating”. Typically, these complaints are preceded by a breakfast or a lunch enriched with simple CHOs. Patients attempt to relieve the symptoms by eating CHO-rich snacks. We propose that this frequent snacking behavior contributes to obesity.

**Figure 4:**
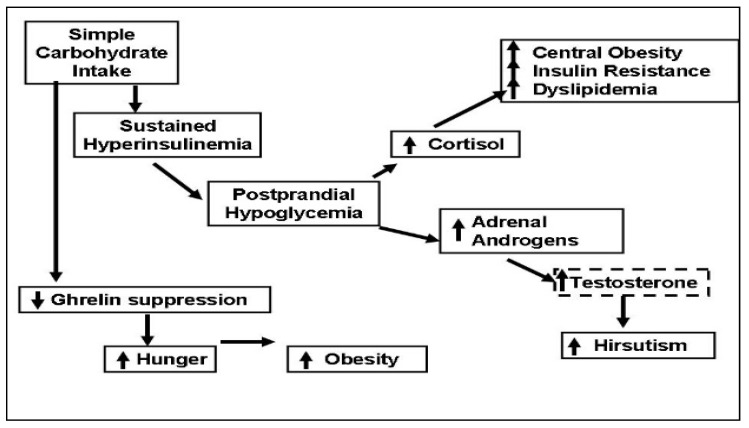
Simple carbohydrate intake can trigger secretion of cortisol and adrenal androgens by causing sustained hyperinsulinemia and postprandial hypoglycemia, which causes sugar craving and frequent snacking. Cortisol secretion leads to central obesity and insulin resistance, and adrenal androgens may worsen hirsutism by converting to testosterone. Simple carbohydrates suppress the hunger signal ghrelin for a shorter period when compared to protein intake, decreasing satiety.

Here, we present a unifying concept linking simple CHO intake to reactive hypoglycemia and hunger, which in turn leads to frequent snacking and obesity (Figure 4).

The following mechanisms are proposed:

1. Polycystic ovary syndrome is an insulin-resistant state, compensated for by the body via hyperinsulinemia. Simple CHO intake results in large amounts of insulin secretion. Insulin levels in the circulation remain elevated after emptying of the stomach, and this causes reactive hypoglycemia. Protein intake also stimulates insulin secretion but does not cause hypoglycemia, possibly because protein intake stimulates glucagon, which facilitates glycogenolysis in the liver [34]. In addition, amino acids, especially alanine, serve as glucose precursors for gluconeogenesis [35].
2. Reactive hypoglycemia triggers an adrenergic response, as evidenced by the symptoms of tremors and sweating and stimulates steroid hormone secretion from the adrenals. Cortisol causes central fat deposition, insulin resistance and dyslipidemia. This concept is supported by our observation that the women with hypoglycemic symptoms were more obese and insulin-resistant. Adrenal androgens are converted to testosterone in the peripheral tissues and increase hirsutism. We found that the women with hypoglycemic symptoms had a 1.7-times higher testosterone level/DHEAS molar ratio as compared to those without hypoglycemic symptoms.
3. Even though protein and CHOs are equally effective in suppressing the hunger signal ghrelin, the effect of CHOs does not last as long. Moran et al. reported no difference between the suppressive effects of oral protein vs. CHO challenges during a 3 h test [36]. We saw that the suppressive effects of CHO vs. protein intakes differed after the 3rd hour. After protein intake, ghrelin remained suppressed for 5 h; after simple CHO intake, ghrelin started to rise by the 3rd hour and returned to baseline by the 5th hour.

In summary, the evidence from our research suggests that simple CHO intake causes reactive hypoglycemia in a significant number of women with PCOS. Reactive hypoglycemia leads to hypoglycemic symptoms and triggers adrenal steroid secretion. In addition, simple CHO intake suppresses the hunger signal for a short time. All these factors may encourage snacking between meals and lead to obesity. Since obesity increases the risk of reactive hypoglycemia, a vicious cycle emerges. Limiting simple CHO intake and increasing protein intake can break this vicious cycle by preventing reactive hypoglycemia and frequent snacking and consequently may improve the anthropometric, endocrine and metabolic outcomes in PCOS.

## Figures and Tables

**Table 1:** Changes in metabolic, inflammatory and endocrine variables (mean ± SEM) before and two months after weight loss program in patients supplemented with either whey protein (*n* = 11) or simple carbohydrate (*n* = 13).

	Baseline	2 mo.	Change	*P*1	*P*2
**Fasting glucose (mg/dL)**					
Whey protein	111 ± 5	110 ± 5	−1.5 ± 3.3	(log)	
Simple carbohydrates	102 ± 6	97 ± 4	−4.5 ± 2.9	0.561	0.513
**Fasting insulin (mIU/mL)**					
Whey protein	31.8 ± 4.2	28.6 ± 4.6	−3.2 ± 2.1		
Simple carbohydrates	23.9 ± 5.0	22.0 ± 5.4	−1.9 ± 2.1	0.664	0.664
**Adiponectin (μg/L) (log)**					
Whey protein	8.6 ± 1.2	6.7 ± 0.6	−1.9 ± 0.8 ^a^	(log)	
Simple carbohydrates	8.6 ± 1.5	8.0 ± 1.2	−0.6 ± 0.8	0.159	0.126
**HOMA**					
Whey protein	9.0 ± 1.3	7.3 ± 1.6	−1.7 ± 1.0		
Simple carbohydrates	6.1 ± 1.8	5.5 ± 1.6	−0.6 ± 0.6	0.306	0.298
**HgBA1 (%)**					
Whey protein	5.7 ± 0.1	5.5 ± 0.2	−0.1 ± 0.1		
Simple carbohydrates	5.4 ± 0.1	5.2 ± 0.1	−0.1 ± 0.1	0.855	0.855
**Triglyceride (mg/dL)**					
Whey protein	149 ± 16	117 ± 19	−32 ± 21		
Simple carbohydrates	92 ± 11	94 ± 12	2 ± 8	0.15	0.0974
**Cholesterol (mg/dL)**					
Whey protein	201 ± 8	168 ± 10	−33 ± 8.4 ^b^		
Simple carbohydrates	164 ± 6	162 ± 7	−2.3 ± 6.8	**0.0089**	**0.0053**
**HDL cholesterol (mg/dL)**					
Whey protein	38 ± 2	34 ± 2	−4.5 ± 1.3 ^b^		
Simple carbohydrates	37 ± 1	36 ± 2	−0.4 ± 1.3	**0.0395**	**0.024**
**Apo B (mg/dL)**					
Whey protein	117 ± 4	97 ± 7	−20 ± 5 ^b^		
Simple carbohydrates	90 ± 5	93 ± 6	3 ± 5	**0.0045**	**0.0045**
**hs-CRP (ng/mL)**					
Whey protein	4.7 ± 0.9	3.7 ± 0.8	−1.0 ± 0.6		
Simple carbohydrates	4.5 ± 1.2	3.6 ± 1.1	−0.9 ± 0.6	0.887	0.881
**Total testosterone (ng/mL)**					
Whey protein	0.95 ± 0.19	0.79 ± 0.11	−0.16 ± 0.16	(log)	
Simple carbohydrates	0.70 ± 0.07	0.73 ± 0.09	0.04 ± 0.04	0.817	0.791
SHBG (nmol/L)					
Whey protein	37.8 ± 8.5	32.7 ± 8.5	−5.1 ± 4.0	(log)	
Simple carbohydrates	48.3 ± 10.6	41.0 ± 7.2	−7.4 ± 6.6	0.774	0.755
**Free androgen index**					
Whey protein	15.8 ± 4.8	14.6 ± 3.0	−1.2 ± 3.9	(log)	
Simple carbohydrates	7.5 ± 1.5	9.2 ± 1.8	1.7 ± 1.2	0.943	0.941
**DHEAS (ng/mL)**					
Whey protein	203.8 ± 34.1	256.8 ± 57.1	53.1 ± 43.8		
Simple carbohydrates	261.8 ± 45.2	275.7 ± 50.6	14.0 ± 22.6	0.425	0.425

Raw data were analyzed. Selected variables were log-transformed to satisfy distributional assumptions. ^a^ *p* < 0.05, baseline vs. 2 months within group. ^b^ *p* < *0*.01, baseline vs. 2 months within group. *P*1: significance of the changes between the whey protein and simple carbohydrate groups based on a 2-sample *t*-test. *P*2: significance of the changes between the whey protein and simple carbohydrate groups based on a mixed-model analysis of variance that adjusts for the baseline values.

**Table 2:** Summary of the intervention studies comparing dietary proteins and simple carbohydrates (WP: whey protein; CHOs: carbohydrate).

	WP vs. Simple-CHO		WP vs. Gelatin	
**Acute Challenge Studies** (Reference [10])	**WP**	**Simple-CHO**		
	***Glucose:*** No change Increased ***Insulin***: Increased Increased ***Ghrelin:*** Suppressed 5 h Suppressed 3 h		-----	
**Adrenal-Steroid Response to Acute Challenge** (Reference [9])	**Responders**	**Non-Responders**	-----	
	***Cortisol:*** Increased No change ***DHEA:*** Increased No change ***Hypoglycemia:*** Yes No			
**Weight Loss studies** Reference [13])	**WP**	**Simple-CHO**	**WP**	**Gelatin**
	***Weight:*** Decreased Decreased less ***BMI:*** Decreased Decreased less ***Fat mass:*** Decreased No change ***Leptin:*** Decreased No change		Similar decreases in weight, BMI, and plasma glucose, insulin, and lipids in both groups	
**Plasma Metabolome Changes** (Reference [18])	------------		Before, but not after, weight loss, branch chain amino acids (Leu, Ile, Val) correlated with homeostasis model assessment of insulin resistance (HOMA) (*p* < 0.05 for all). Proline and cystine related pathways discriminated WP vs. gelatin supplementations.	

## Data Availability

Data were contained in the individual articles.
